# Short-term efficacy of reducing screen media use on physical activity, sleep, and physiological stress in families with children aged 4–14: study protocol for the SCREENS randomized controlled trial

**DOI:** 10.1186/s12889-020-8458-6

**Published:** 2020-03-23

**Authors:** Martin Gillies Banke Rasmussen, Jesper Pedersen, Line Grønholt Olesen, Søren Brage, Heidi Klakk, Peter Lund Kristensen, Jan Christian Brønd, Anders Grøntved

**Affiliations:** 1 Department of Sports Science and Clinical Biomechanics, Research Unit for Exercise Epidemiology, Centre of Research in Childhood Health, University of Southern Denmark, 5230 Odense, Denmark; 2 MRC Epidemiology Unit, Cambridge School of Clinical Medicine, Institute of Metabolic Science, University of Cambridge, Box 285, Cambridge Biomedical Campus, Cambridge, CB2 0QQ UK; 3 Department of Physiotherapy and Research Center for Health Science, University College Lillebælt, Odense, Denmark

**Keywords:** Screen time, Physical activity, Sleep, Stress, Randomized controlled trial

## Abstract

**Background:**

During the recent decade presence of digital media, especially handheld devices, in everyday life, has been increasing. Survey data suggests that children and adults spend much of their leisure on screen media, including use of social media and video services. Despite much public debate on possible harmful effects of such behavioral shifts, evidence from rigorously conducted randomized controlled trials in free-living settings, investigating the efficacy of reducing screen media use on physical activity, sleep, and physiological stress, is still lacking. Therefore, a family and home-based randomized controlled trial – the SCREENS trial – is being conducted. Here we describe in detail the rationale and protocol of this study.

**Methods:**

The SCREENS pilot trial was conducted during the fall of 2018 and spring of 2019. Based on experiences from the pilot study, we developed a protocol for a parallel group randomized controlled trial. The trial is being conducted from May 2019 to ultimo 2020 in 95 families with children 4–14 years recruited from a population-based survey. As part of the intervention family members must handover most portable devices for a 2-week time frame, in exchange for classic mobile phones (not smartphones). Also, entertainment-based screen media use during leisure must be limited to no more than 3 hours/week/person. At baseline and follow-up, 7-day 24-h physical activity will be assessed using two triaxial accelerometers; one at the right hip and one the middle of the right thigh. Sleep duration will be assessed using a single channel EEG-based sleep monitor system. Also, to assess physiological stress (only assessed in adults), parameters of 24-h heart rate variability, the cortisol awakening response and diurnal cortisol slope will be quantified using data sampled over three consecutive days. During the study we will objectively monitor the families’ screen media use via different software and hardware monitoring systems.

**Discussion:**

Using a rigorous study design with state-of-the-art methodology to assess outcomes and intervention compliance, analyses of data from the SCREENS trial will help answer important causal questions of leisure screen media habits and its short-term influence on physical activity, sleep, and other health related outcomes among children and adults.

**Trial registration:**

NCT04098913 at https://clinicaltrials.gov [20-09-2019, retrospectively registered].

## Background

Time spent using screen-based media devices is ubiquitous in everyday life of children and adults of the twenty-first century. Rapid technological development and market introduction of handheld screen-based devices, such as smartphones and tablets, to consumers all over the world, has changed the way and the amount of time humans interact with electronic media. To the extent that self-report depicts screen time habits accurately, evidence suggests that British children and youth (8–18 years) engage in four hours and 45 min of screen time a day on average, as a main activity or while engaging in other activities [1]. Furthermore, results from the same study indicate a pronounced increase in screen time from 2010 to 2015 in British children [1] and an increase in computer use during leisure hours from 2001 to 2016 in most age groups in North America [2] has been reported. Based on a 2018 survey of 3660 school children in Denmark, 24% of boys and at least 19% of girls aged 13 and 15 spend at least four hours/day on weekdays watching movies, tv-series, Youtube-movies or entertainment shows [3]. Also, 88% of adult Danes report using the internet daily, as part of their daily routine [4]. Clearly, adults and children spend much of their leisure time engaging in some form of entertainment-based screen media.

In the public debate, there is much discussion about whether use of screen media carries a risk to our mental well-being and physical health. According to a 2016 Technical report from The American Academy Pediatrics, screen-based media use includes some beneficial effects, such as improved knowledge acquisition at an early age, access to important information and creating enhanced opportunities for communication [5]. However, there is also evidence which suggest that screen media use has a negative relationship with children and adolescents’ sleep [6], as well a myriad of other aspects of health, including adiposity, unhealthy dietary pattern, symptoms of depression, a poor quality of life [7] and decreased physical activity [8]. Of concern may be the effect of excessive screen time in childhood on children’s physical activity habits. Some evidence suggests that childhood physical activity habits track to some degree into young adulthood [9] and concerns may therefore be raised regarding lifelong physical inactivity.

Since just before the turn of the century, experimental studies have been conducted to investigate the effect of change in screen media use on health-related outcomes in children. Among the randomized controlled studies, which have investigated the effect of a reduction in screen media use on physical activity [10–17], only one found significant increase [14]. However, this trial was limited by measuring physical activity by self-report [14]. This and several of the other randomized controlled studies are limited by small samples sizes [10, 11, 13, 14] and only one trial measured change in screen time via an objective instrument [15]. Furthermore, only one study emphasized the impact of a screen media reduction on change in physical activity in adults (the primary caregiver) [12].

In addition to the effect of screen time on physical activity, some recent lab-based experimental evidence suggests that exposure to digital screens may also negatively affect circadian rhythm and sleep in adults [18–20]. Although some evidence suggests an impact of limiting screen-based media use on health, several of the studies include noteworthy methodological limitations. Therefore, we are still limited in our basic understanding of the causal relation between screen media use and physical activity, sleep and physiological stress. Furthermore, because of staggering screen media technology changes in the past decade, some existing research on screen media use may have limited generalizability to current screen time behavior and culture. Rigorously conducted randomized controlled trials in free-living settings including adults and children, employing objective measures to detect changes in both exposure and outcomes, are needed to refute or confirm hypotheses of how habitual use of screen media, in its modern form, affects physical activity, sleep and physiological stress. The SCREENS trial is a randomized controlled trial which aims to investigate the short-term efficacy of limiting leisure screen media use on objectively assessed habitual physical activity, sleep duration and quality, in parents and their 4–14-year old children and measures of physiological stress, in adults.

### Objectives

The objectives of the SCREENS trial are to investigate the short-term efficacy of limiting screen media use during leisure on adults’ and children’s:

- Non-sedentary time (all activities not performed in a sitting or lying position) during leisure measured by combined hip- and thigh worn accelerometry
- Total sleep time, sleep latency, and wake after sleep onset measured by home-based single channel electroencephalography (EEG) sleep monitoring
- Parent reported psychological well-being, in their children
- Leisure- and total time engaged with moderate- and vigorous physical activity

Also, in adults, the objectives of the study are to investigate the short-term efficacy of limiting screen media use during leisure on:

- Subjectively assessed sleep quality
- The cortisol awakening response and diurnal cortisol obtained from saliva sampling, as markers of physiological stress
- Heart rate variability using 24-h assessment, also as a marker of physiological stress
- Self-reported mental well-being and mood states

The study tests the hypotheses that restricting leisure screen media use to an amount much below habitual levels for a period of two weeks increases leisure time spent being non-sedentary, increases total sleep duration and decreases markers of physiological stress, in families of adults and children.

## Methods/design

### SCREENS pilot trial

From November of 2018 to March of 2019 we conducted the SCREENS (not an abbreviation) pilot trial (ClinicalTrials.cov ID: NCT03788525) in families residing in the Municipality of Middelfart on the Island of Funen, in Denmark. The purpose of the pilot was to assess degree of compliance to the prescribed intervention, compliance to home-based objective assessments of physical activity, sleep, and measures of physiological stress. The purpose was furthermore to assess feasibility of our recruitment strategy, as well as resources required of participants and researchers involved and other general aspects of conducting such a study, previously outlined in detail [21]. Preliminary results from the pilot study show that the measurement and intervention protocol generally were feasible, although adjustments prior to conducting the full-scale randomized controlled trial of the SCREENS trial, were necessary. These adjustments are included in the protocol for the full-scale SCREENS randomized controlled trial described in this paper.

### SCREENS survey and randomized controlled trial

This study was commenced when a survey was sent out medio May 2019 to selected postal districts in the Municipality of Odense. The study is now being expanded to the remaining municipalities on Funen (except Middelfart). The following sections will describe in detail the methodology of the SCREENS trial, including a description of the recruitment processes based on a population-based survey. A home- and family-based screen media use reduction intervention will be evaluated using a two-arm, parallel, randomized controlled superiority trial design. This study protocol was developed in accordance with the SPIRIT 2013 checklist for study protocols of randomized controlled trials (see Additional file 1).

### Recruitment process

**Fig. 1:**
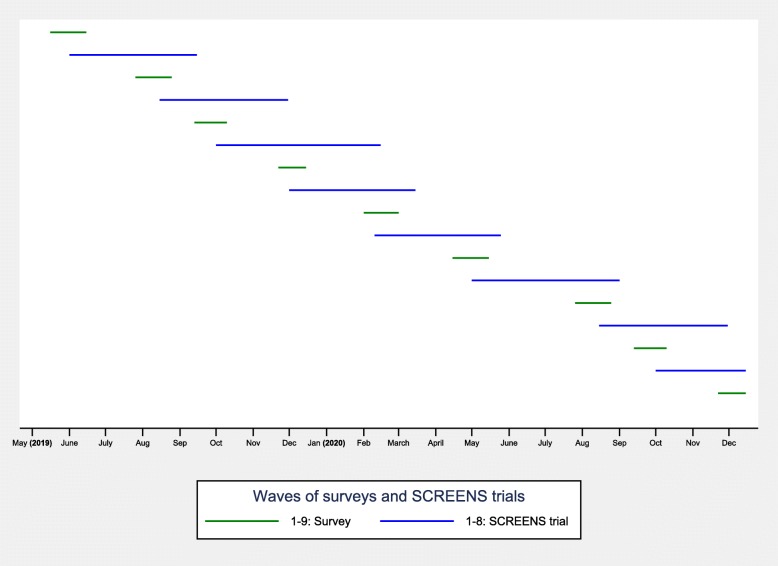
Overview of surveys and subsequent recruitment for and conduct of the SCREENS trial. A visual overview of the approximately one-and-a-half-year span of the study, which includes digitally mailing out surveys including questions regarding screen media use in children and adults. Following each survey is recruitment for and conduct of the SCREENS trial. The designation of each month on the x-axis denotes the first day of said month. Notice that the duration and timing of each wave (survey and experiment) varies, as some of the depicted waves include periods without any activity because they span holidays. However, for the sake of simplicity, this has not been changed

The recruitment consists of two stages; the first stage is sending out a survey to approximately 3000 Danish adults residing in the municipality of Odense (the fourth largest Danish municipality) or neighboring municipalities, via an electronic mailbox system (e-boks) available to Danish citizens. There is a dual purpose of the survey; first, to obtain extensive descriptive data on modern screen media behavior among adults and children 6–10 years of age, and secondly, to serve as a recruitment platform for the SCREENS trial. Survey respondents will at the end of the survey be invited to hear more about the SCREENS trial. The second stage include contacting families who have responded to this invitation. Stage 1 and stage 2 will be repeated, in the different municipalities. Adults whose families are eligible are invited to participate in the trial. A broad overview of the survey send outs and recruitment for and participation in the SCREENS trial is given in Fig. 1.

### Stage 1: Recruitment via survey

**Fig. 2:**
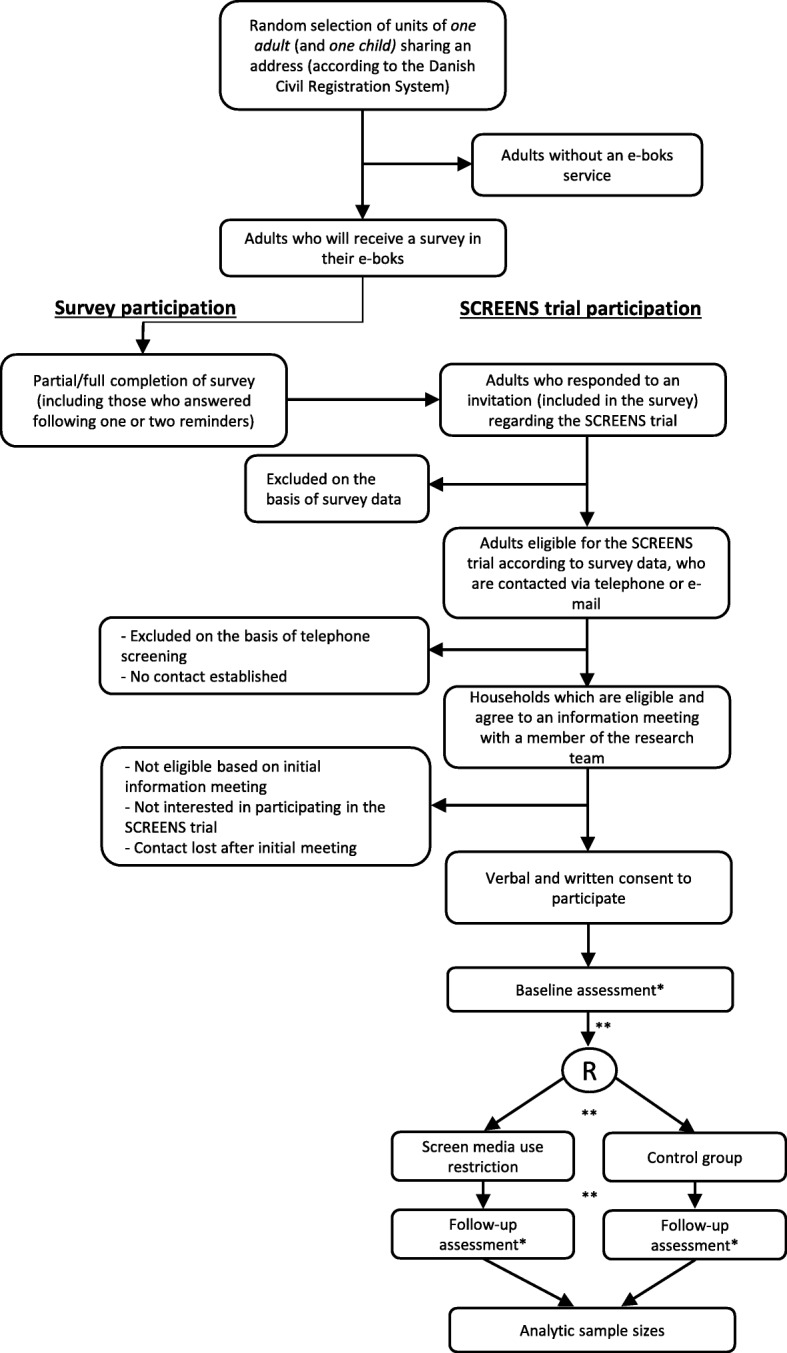
Flow chart of participants from recruitment to statistical analyses. The flow chart above gives a broad overview of the recruitment processes via an electronic survey, initial phone contact, meeting in the families’ household, participation in the SCREENS trial and, ultimately, the statistical analyses. R; Randomization, *; Possible source of missing data, **; Stages at which participants may choose to discontinue

Figure 2 gives an overview of the flow of participants through the study in its entirety. For the survey, one randomly selected adult and one randomly selected child household member between six and ten years of age will be invited. To receive the survey, the adult and the child must share an address and the adult must have full custody of the child, according to the Danish National Civil Registry. No further restrictions are put on the invitees of the survey and thus all households in the municipalities that meet the criteria above will be invited. The sampling frame of the survey invitees was gathered from the Danish National Civil Registry obtained through the National Health Data Authority. The survey includes questions for the adult pertaining to the adult’s and the child’s screen media habits, including amount of screen use and questions on other domains of the family screen media home environment, such as rule setting.

Respondents are asked in the survey whether they would like to be contacted regarding a different study (the SCREENS trial). Therefore, all potential participants of the SCREENS trial will be survey respondents. Those who answer ‘yes’ to the question in the survey and who meet the following preliminary inclusion criteria (based on survey questions) will be contacted via phone regarding participation:

- The adult must be classified as having high screen media use, which we define as being above the 40th percentile for total screen time during leisure, according to a questionnaire battery, included in the survey. The 40th percentile was estimated as 2 h and ~ 23 min/day (weighed average of week- and weekend days) based on the first 1000 survey respondents (all from the Municipality of Odense). This arbitrary cut-point was defined as a compromise between assuring enough eligible adults such that recruitment into the study would progress at a reasonable pace, whole making sure to include only those with enough screen media use during leisure. This criterion was based only on the adult who completed the survey, as arguably his or her screen media use to some extent could be a proxy for the screen media use of the entire family.
- To exclude families who are coping with e.g. disturbed sleep patterns and others stress factors from having either newborns, toddlers or very young children, households must include only children ≥4 years of age.
- Adults must not be outside the labor market or educational system.
- Adults must not have any regular night shifts.

Recruitment via surveys will continue until we reach the number of participants required for the statistical analyses (see “Justification for sample size”). We expect to have sampled enough participants for the trial following the 8th or second last survey. Thus, in the final survey, we expect to not include the question regarding the SCREENS trial (see Fig. 1, to the right).

### Stage 2: Recruitment following survey

Those respondents who meet the initial criteria will receive a phone call from a member of the research team who will explain the content of the SCREENS trial. Further screening will take place in two steps; first, on the phone, we will screen for health-related matters and practical issues regarding the project. The adult must confirm that:

- At least one adult and one child, from the household, is willing to participate
- The family has the resources, primarily in terms of spare time, to complete the study as outlined. This includes being able to restrict leisure screen time, including in weekends, during a two-week timeframe
- At least one participating adult and all participating children must be able to hand-over their smartphone(s) and tablets for the screen time restriction period
- The family is motivated to restrict leisure screen time for a short period of time

These exclusion criteria for both adults and children include:

- Not being able to engage in regular physical activity during everyday life
- Having a diagnosed sleep disorder that continues to affect sleep
- Diagnosed with or in the process of being cleared from any neuropsychiatric disorders, such as attention deficit hyperactivity disorder, or developmental disorders, such as autism spectrum disorders
- Been on sick leave within the last 3 months due to stress

If families are eligible according to the criteria above, a mandatory information meeting of approximately 45 min will be held at the family’s home. Here, the study will be explained in detail, also including a demonstration of the measurement equipment used in the study. At this meeting we will also register the amount of screen media units available in the household. All families will be offered a minimum of 24 h to consider whether they would like to participate. Families are handed a written consent form, which must be filled out, before participation in SCREENS trial. Figure 2 below provides a complete overview of the flow of participants through the study in its entirety.

### ‘Active’ and ‘passive’ participants

Based on our experience from the SCREENS pilot trial, some families choose or are only able to include some family members in the study. Those who participate we define as ‘active’ participants. Conversely, members of the household who do not take part in the study are defined as ‘passive’ participants. If a family chooses to include only partial participation of the household, it is an inclusion criterion that ‘passive’ participants fully support the constraints of the 2-week screen time restriction period that ‘active’ participants in the household must comply with. Teenagers between 15 and 17 years are by default assigned to be ‘passive’ participants. ‘Passive’ participants are not required to hand-over their smartphone(s) and tablet(s).

### Participant safety

As the authors are not aware of any side-effects associated with participation in the study, the study participants are informed verbally and in writing that they should notify the researchers if they experience any side-effects or harm during the SCREENS trial. No specific protocol to mitigate adverse events has been developed and there has not been formulated a formal trial termination procedure. All study participants are informed verbally and in writing that they can withdraw their participation from the study at any time, without any need to justify their reason for doing so.

### Justification of sample size

The primary outcome is average change in accelerometry-derived non-sedentary time (min/day) during leisure, in children. Based on families, who currently either have or are registered for the SCREENS trial, we expect to include 1.96 children per family. Based on preliminary data from our pilot, we expect a standard deviation of 57 min/week for average change in non-sedentary time from baseline to follow-up in the experimental group and 39.7 min/week for the control group. Based on other internal work in children 0–17 years of age, we expect a 0.3 correlation between sibling non-sedentary time. In regard to clinical relevancy, a cross-over study found that intermittent interruptions of walking, amounting to a total of 18 min during 3 hours of sitting, resulted in favorable metabolic changes, when compared to sitting only, in children 10.2 (1.5) years of age [22]. For the current study, a 24-min change is deemed a clinically relevant effect size, based on the expected between-group difference and project resources. Therefore, assuming an intraclass correlation coefficient of 0.3 for sibling non-sedentary time and a cluster size of 1.96 children per family, to detect a 24 min/day difference with a power of 80% and α = 0.05, a total of 88 families including 174 children is required for the analyses.

Thus far we have experienced a 0 % drop-out. Therefore, the main threat against achieving enough subjects for our analyses may arguably be missing data. Therefore, by sampling 95 families into the study, including a total of 186 children, we have safeguarded our primary analysis against a family dropout rate or data loss for other reasons of approximately 7.4%.

### Organization of the SCREENS trial

The study is organized into a planning/working group and a data collection group. The former consists of the entire list of authors, whereas the latter consists of MGR, JP, as well as pre-graduate student SS, and student helpers Stud. Cand. Scient. SM and Stud. Cand. Scient JH.

### Study design and structure of the SCREENS trial

**Fig. 3:**
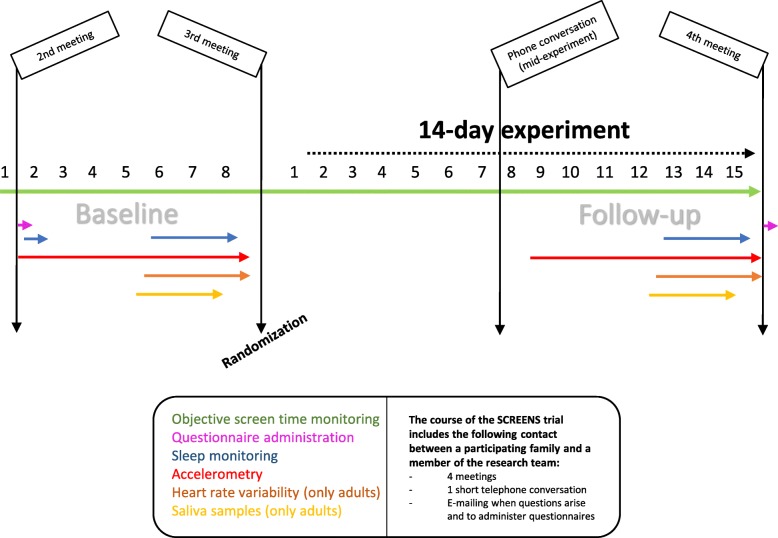
An overview of the SCREENS trial as well as the included measurements. The figure illustrates the course of the SCREENS trial scaled in days, including the experiment phase and the timing and duration of each outcome measurement protocol. Notice that the protocol for baseline measurements and the protocol for follow-up measurements, differ only in that there is one additional day of sleep measurement at baseline (a “test” night to get acquainted with this protocol) and that the questionnaires are administrated at opposite extremes. The first meeting is an information meeting in the families’ household, whereas the second through fourth meeting take place during the trial

Figure 3 gives an overview of the course of the SCREENS trial, as well as the exposure and outcome measurements. As illustrated, the SCREENS trial includes three meetings and one phone call, which takes place in the following chronological order; a baseline meeting, a post-baseline/pre-experiment meeting, a mid-experiment/pre-follow-up phone call and a post experiment/post-follow-up meeting. The meetings will all take place in the families' household and will be held by the same member of the research staff. The baseline and follow-up periods last a week (7 × 24 hours) and span 8 days. If e.g. a period starts at 5 pm on a Wednesday, the period finishes on the same day and at the same time, exactly a week later.

#### Intervention

The intervention takes place in the families’ household during everyday activities. The intervention requires that the family make several changes during everyday life, regarding screen time habits, during leisure, for 2 weeks. Two weeks was chosen as a compromise between assuring that there was enough time to detect change (including time to adapt), with how long we could expect families to heavily restrict leisure screen time. One of the main components of the intervention is that portable screen-based devices (smartphones and tablets) must be handed over to the research staff for the duration of the intervention. The devices will be stored in a locked safe at the Department of Sports Science and Clinical Biomechanics at the University of Southern Denmark. Every family member who owns a device with a sim card will be offered a Nokia 130 phone in exchange, in which their sim card will be inserted. The Nokia phone can perform the few operations that arguably are essential during everyday life; calling, text messaging, and setting alarm clocks.

During the screen media reduction period, which only targets leisure hours, a finite amount of entertainment-based screen media use is allowed. Entertainment-based screen time is defined as; watching streaming-based services (Netflix, HBO, Amazon, Youtube etc.), most broadcast television, surfing online, gaming, use of social media to connect with friends and family and more. In the context of this study, it also includes watching the news (although it may be debatable whether this is considered entertainment-based). During the 2-week screen time restriction, adults and children are allowed up to 3 hours per week per person of entertainment-based screen media use. On the contrary, *necessary* screen time includes brief contact via phone to plan social gatherings, including social arrangements with one’s family or play dates for one’s children, as well as necessary shopping online, e.g. grocery shopping. Necessary screen time also includes all screen time relating to one’s children’s school or nursery, e.g. reading information letters from the teacher. Adults are permitted up to 30 min of necessary screen time per day. Children or adolescents, who are required to do homework on digital screens, are permitted to do so with no constrains on time. All entertainment-based and necessary screen time must be noted in sheets that will be handed out at the post-baseline/pre-experiment meeting.

The research staff will place three to five ‘intervention reminders’ in the household. An ‘intervention reminder’ is a A5 sheet on which the rules of the intervention are listed. These will act as environmental cues, where the goal is that when family members see the reminder they are reminded of the details regarding their participation in the study. The reminders will be placed strategically in the household, including in the living room by the television, at household computers and in a place where the family often gathers, e.g. in the kitchen. Table 1. gives a summary of the core components of the screen media restriction protocol.

**Table 1:** Summary of core components of the screen media reduction protocol

Core components	Content
Planning the course of the SCREENS trial	Careful scheduling of the trial between researcher and family, such that the 2-week restriction in screen time is possible
Installation of screen time monitors on household devices	Installation of software or hardware on the screen-based devices in the household, including televisions, computers and portable devices (smartphones and tablets)
Encouraging the family to plan for 2-week screen time break	The family will fill out a form where they note potential challenges and solutions to challenges during a 2-week screen time break
Handing over portable screen-based media devices	At least one participating adult and all participating children must hand over their portable screen-based devices (smartphones and tablets)
Restrictions on leisure entertainment-based screen time	Family members must limit their entertainment-based screen time during leisure hours to ≤3 h/week/person
Allowing for some necessary screen time	Adults are allowed no more than 30 min per day of necessary screen time, during leisure hours. To the extent that it is necessary, young adults can do their homework with no limit in terms of time. All necessary screen time must be noted.
Registration of all screen time during experiment phase	The family members must register all screen time, entertainment-based and necessary, during the experiment phase, on paper sheets
Placing three to five ‘intervention reminders’ in household	One in living room on television(s), one at a computer and one in a place where the family often gathers
Financial incentive upon completion of the study	Each family receives 500 DKK upon completion of the study

The table above gives an overview of the core components of the SCREENS trial and includes a short description of what these each entail

The goal is to intervene as little as possible in the potential behavior change of the family, beyond establishing the framework of the intervention. The purpose of this is to increase as much as possible the ecological validity of the data.

The control group will continue with their everyday habits, only interrupted by follow-up measurements. Those who are randomized to the control group will be offered to complete the intervention protocol when they have finished the control group period, although they will not repeat the outcome assessments a third time.

#### Scheduling meetings

All meetings and phone calls will be scheduled in advance of the study. This is to ensure that meetings and measurements are structured in accordance to the overall protocol. This includes making sure that baseline and follow-up periods are placed at approximately the same days during the week (to ensure comparability). Lastly, early planning is also to ensure that the 2-week screen time restriction is placed at a time where the family can manage to implement the behavior change. Ideally, the course of the trial should be scheduled in a manner, such that baseline and the experiment phase, including follow-up measurements, are placed back to back. However, for the latter point, for practical reasons regarding the family’s schedule, it may be necessary to allow a week or more to pass between end of baseline and the beginning of the experiment. Reasons for doing so could be that a family is travelling at this time or if there are upcoming television events, which the family would like watch, e.g. national soccer events, which collide with an experiment phase scheduled at an ideal time. Judgements regarding scheduling conflicts will be considered weighing the importance of assuring progress of the trial against pragmatic considerations.

#### Baseline meeting

At the baseline meeting, the research staff will instruct the families on how to conduct the protocol for collecting data. Because the families oversee data collection themselves, extensive training will be given regarding the protocols. At this meeting, the family members will be instructed start wearing accelerometer belts and we will also demonstrate the use of the sleep equipment (details on outcome measurement protocols later).

At the meeting we will install instruments to objectively assess screen time on the devices in the household in attempt to accurately monitor compliance. We will install an application (SDU Device Tracker) on the family members’ smartphones, and tablets. Furthermore, we will mount tv-monitors on every TV in the household. Finally, tracking software for PCs will be installed on every PC in the household, if possible (the monitoring systems are described in more detail, later).

At the end of the meeting a sheet will be handed out, which outlines some of the practical matters regarding the intervention. The sheet also includes several points for discussion in the family, as they are encouraged to discuss what challenges a 2-week screen media use reduction period could entail. They are also encouraged to discuss how any issues might be resolved. This includes a discussion of how to manage everyday tasks without a smartphone at one’s disposal. The family members are encouraged to write down these challenges and their potential solutions.

#### Post-baseline/pre-experiment meeting and randomization

At the post-baseline/pre-experiment meeting the family will be randomized by research staff using the Odense Patient data Explorative Network (OPEN) Randomize platform (part of a research infrastructure service for researchers in the Region of Southern Denmark), to either the screen media restriction protocol or to control. Staff at the OPEN Randomize platform - whom the research staff do not work with - have generated a randomization table with a hidden allocation sequence using computer generated random permuted blocks of 2–4 families and an allocation ratio of 1:1. After randomization the group allocation will not be masked to the research staff and given the nature of the intervention cannot be concealed from the participants.

At this meeting, we will also handover new accelerometers belts for the follow-up measurements. All other equipment can be re-used.

#### Mid-experiment/pre-follow-up phone call

Between the post-baseline/pre-experiment meeting and the post experiment/post-follow-up meeting, the research staff will phone the contact person in the family. The purpose of this phone call is twofold; first, to motivate the family to maintain the screen time reduction during the entire 2-week duration. The researcher will ask the family how well they are doing in terms of restricting their screen time according to the protocol. Secondly, the purpose of the phone call is to remind the family that they must soon start up the follow-up measurements and to ask the family if they have any questions relating to the measurement protocol. The control group will also receive a phone call, only for the second purpose.

#### Post experiment/post-follow-up meeting

At the post experiment/post-follow-up meeting, which takes place immediately or shortly after the end of the experiment phase, the researcher will congratulate the family on their completion of the study. The children will each receive a colorful diploma signed by the member of the research staff in charge of this family. The families will receive a 500 DKK reimbursement for the time they put into completing the study (independent of group allocation).

### Theoretical underpinning of intervention

Bandura’s social cognitive theory [23] serves as the theoretical framework of the intervention. The reciprocally determined and causal relationship between an individual’s environment, his or her personal factors and behavior, as proposed by Albert Bandura [23], serves as a theoretical underpinning of the core components outlined earlier. Specifically, we target personal factors and induce specific changes to the household environment, to encourage the families to change, i.e. decrease the level of their screen time. Conversely, we expect that changes in screen media use may also influence personal factors, such as an individual’s biology, as defined by Bandura [23], e.g. sleep quality, mood, and mental stress.

We specifically target the families at the personal level through several means. At an early stage in the trial, we ask the families to discuss and find solutions to issues they may meet during a 2-week screen media use reduction. Although the researchers do not get involved in the details, we encourage planning and goal-setting [24]. The study is emphasized as family-based and therefore we encourage a change in the overall household culture relating to screen media use. We suggest that parents act as models of this new behavior, which might make it easier for the children to adopt the behavior, as well [25].

Meeting only one research staff member might increase the level of commitment to the intervention protocol, via increased self-regulation [26] in terms of decreasing screen media use. This may arguably be because one knows one must face the same member of the research team several times during a short time period.

During the 2-week restriction period we make specific changes to the household environment; we install monitoring systems on the household televisions and computers, as well as on smartphones and tablets. We also place three ‘intervention reminders’, i.e. environmental cues. The goal of all these environmental changes is to emphasize self-regulatory processes [26] necessary to change and maintain the screen-time reduction. The mid-experiment phone call is included for a similar purpose.

The goal of the reimbursement following completion of the study is to have an incentive structure, an extrinsic reward, i.e. an reward coming from something exterior to the person [27]. By anticipating a reward, this may facilitate the self-regulatory processes [26] required to be compliant to the SCREENS trial protocols.

Below is a table summarizing the elements of Social Cognitive Theory discussed above (see Table 2). The table is structured with inspiration from Table 1 in the study by Hinkley et al. [13] aiming to reduce screen media use in 2–3-year-old children.

**Table 2:** Bandura’s Social Cognitive Theory (SCT) as a theoretical framework for the intervention

Screen media restriction components	SCT level targeted	Behavior element targeted
Planning the course of the screen time restriction period. Hanging notes where family members can see them.	Personal / environment	Goal setting, self-regulation
Meeting the same individual from the research team during several meetings throughout the SCREENS trial	Personal	Self-regulation
Emphasizing the project as a *family* project, including parents as role models	Personal and environment	Modelling
Installation of screen time monitors on household devices	Environment	Self-regulation
Placing three to five ‘intervention reminders’ in the household	Environment	Self-regulation
Motivation and encouragement midway through the two-week screen time restriction	Personal	Self-regulation
Financial incentive upon completion of study	Personal	Extrinsic reward, self-regulation

The table above gives an overview of the how the components of the screen media restriction part of the SCREENS trial are theoretically grounded in Social Cognitive Theory. We outline which level that is targeted and which specific behavioral theory which is targeted at this level

### Exposure and outcome measurement protocol

As already mentioned, figure three gives an overview of the overall trial protocol, including an overview of the timing of measurements. On the first day of baseline measurement, questionnaires will be administered to the participants. The questionnaires will be sent by e-mail to an adult in the household. Then, on the first night, the first of four sleep measurements during baseline will be carried out. The first sleep measurement is meant as a “test” measurement (not included in statistical analyses and not included at follow-up), as some adjustment to the sleep measurement protocol is necessary. The three remaining consecutive sleep measurements will take place from the night of day five till day six through the night of day seven till day eight. On day one, accelerometers will be mounted onto the participants and worn throughout the given measurement period. On day four, adult participants will be instructed to mount heart rate variability monitors to their upper body at the same time accelerometers were mounted 4 days prior. These monitors will be worn for three consecutive days. Therefore, accelerometry and heart rate variability measurements will finish on the exact same time on day eight. Finally, from the morning of day five till the evening of day seven, adult participants will collect four saliva samples per day; three in the morning upon awakening and one before bedtime.

### Primary and secondary outcomes and endpoints

The primary outcome is the mean between-group difference in accelerometry-derived non-sedentary time (min/day) during leisure, in children. The primary endpoint is the primary outcome at follow-up assessment (only one follow-up).

The full list of secondary and exploratory outcomes can be found at: https://clinicaltrials.gov/ct2/show/NCT04098913.

### Accelerometry

Both adults and children will undergo 24-h accelerometry for seven consecutive days using two Axivity AX3 (Axivity Ltd., Newcastle upon Tyne, United Kingdom) triaxial accelerometers. The sensors are small (dimensions: 23 mm × 32.5 mm × 7.6 mm) weighing only 11 g. Acceleration is measured in three axes. Sensitivity will be set to +/− 8 g and sampling frequency to 50 Hz.

The accelerometers are worn at two anatomical locations; one is fixated to the body in a pocket attached to a belt worn around the waist, where the sensor is placed such that it is on the right hip facing away from the right of the body. A second belt is worn around the right thigh midway between the hip and the knee, where an accelerometer is placed in a pocket on this belt facing away from the body. The devices will be worn for 1 week (seven consecutive days) at baseline and at follow-up, which has been suggested as the number of days required to estimate habitual physical activity [28].

Time spent in distinct activity types (sitting, moving, standing, biking, stair climbing, running, walking, and lying down) are determined from the acceleration measured with the thigh worn device using the method proposed by Skotte et al. (2014) [29] using 1-s epochs. In this study, the method was validated with adults in a standardized field test and demonstrated a sensitivity > 95% and specificity > 99% for all activities. Also, during almost 6 days of measurement in free-living, sensitivity and specificity were 98 and 93%, respectively, for classification of sitting time [29]. Children and adolescent specific decision thresholds for the method were developed using an internally conducted study (publication in preparation). The results indicate high sensitivity and specificity of measurement. Non-sedentary time is defined based on this method and includes all activities, including standing, other than sitting and lying. We will analyze non-sedentary time as the amount per day (total amount per 7 days divided by seven). In addition, time spent within physical activity intensity domains (sedentary, light, moderate and vigorous) will be estimated using ActiGraph counts generated with the waist worn device [30] using 10-s epochs. The cut-points defining intensity domains are determined using an internal calibration study conducted in a group of children and adults (results not published).

Non-wear periods are identified and marked as missing data by evaluating three signal features generated from acceleration in combination with temperature and predefined expected awake time (06:00 AM to 10:00 PM). Periods of no movement (acceleration below 20 mg) longer than 120 min are always identified as non-wear and shorter periods from 45 to 120 min are identified as non-wear if the average temperature is below an individually estimated non-moving temperature (NMT) threshold. Periods of 10 to 45 min with no movement are only identified as non-wear if the average temperature is below the NMT threshold and if the end of the period is within the expected awake time. Device transportation (periods of device movement when the device is not worn by the subject) is identified as non-wear if the average temperature of the period is below the NMT threshold.

A valid day (restricted to leisure time during wake hours) of measurement will be defined as a day containing no more than a total of 10 % non-wear. A valid baseline and follow-up measurement must include at least three valid weekdays and at least one valid weekend day. We will include a sensitivity analysis of the valid data where we impute for each individual non-wear time based on available data, for the same type of day and timing of non-wear (time of day).

The software OmGUI version 1.0.0.37 will be used in the set-up, download, re-sampling and conversion of the accelerometer data. The raw accelerometry data will be processed using Matlab (Mathworks Inc., Natick, Massachusetts, US) release R2019a version 9.6.0.1099231.

#### Sleep monitoring

Both adults and children will undergo sleep assessments using the Zmachine® Insight+ model DT-200 (General Sleep Coorporation, Firmware version 5.1.0). The device measures sleep by single channel electroencephalography (EEG) from the differential mastoid (A_1_–A_2_) EEG location on a 30-s epoch basis. The sleep apparatus is developed for use in a free-living setting for objective measurement of sleep, including measurement of sleep duration and sleep stage classification (Light Sleep (N1 & N2), Deep Sleep (Slow Wave Sleep) and Rapid-eye movement (REM) Sleep), as well as computation of sleep-specific quantities, e.g. latency to the respective sleep stages. The algorithm in Zmachine insight+ has been compared to polysomnography (PSG) in adults with and without chronic sleep issues within a laboratory setting and shown a high degree of validity for this purpose [31, 32]. The Zmachine device has also been shown to be valid in terms of classification of sleep; detecting of sleep versus awake with the Zmachine algorithm was done with a sensitivity, specificity, positive predictive value and negative predictive value of 95.5, 92.5, 98 and 84.2%, respectively against polysomnography [31]. A second Zmachine algorithm, which further differentiates sleep into specific stages, has also been evaluated; classification of sleep into light, deep and REM was achieved with sensitivities of 84, 74 and 72% and with positive predictive values of 85, 78 and 73%, respectively against polysomnography [32].

Three electrodes (Ambu A/S, type: N-00-S/25) are mounted on the mastoids (signal) and the back of the neck (ground). Thirty minutes before the subjects plan to go to bed to sleep, the skin areas are cleansed with an alcohol swab and then electrodes are attached to the skin. An EEG-cable connects the three electrodes to the Zmachine device, whereafter a sensor check is performed to detect whether one or more electrodes are not mounted correctly. If there are sensor problems, these are solved swiftly by a simple change of said electrodes.

Because the Zmachine device has not been developed for children directly and because some adults may tend to twist and turn during sleep, we developed custom-made pockets, which allow for fixation of the EEG-cable and Zmachine device itself to the accelerometer belt at the hip. This fixation assures e.g. that cables will not wrap around the child’s neck during sleep. We also recommend these pockets for adults whose sleep includes much twisting and turning.

#### Mental stress: heart rate variability, cortisol awakening response and diurnal cortisol

In adult participants, physiological stress will be assessed by measuring three components associated with human stress; the beat-to-beat interval variability (ms) of the heart [33], the saliva cortisol awakening response (a unique feature of cortisol circadian rhythm) [34] and 24-h diurnal saliva cortisol [35]. As habitual excessive screen time may negatively impact both main stress pathways – the sympathetic adrenal medullary (SAM) axis and the hypothalamic pituitary adrenal cortex (HPA) axis [36] – we include multiple measurements of physiological stress.

#### Heart rate variability

We will collect 24-h measurements of Heart Rate Variability (HRV) for three consecutive days using the Firstbeat Bodyguard 2 HRV measurement device. The device is non-invasive and allows ambulatory continuous recording of R–R heartbeat intervals in a free-living setting. In addition, after merging and aligning epoch-by-epoch data on intensity and type of physical activity from accelerometry with R-R beat intervals, HRV activity arising from physical exertion will be delineated from non-physical activity associated HRV activity. The latter is proposed to be reflective of states of mental stress. A recent systematic review and meta-analysis provide evidence for construct validity of HRV as an objective measure to quantify psychological stress [33].

Adults will mount the Firstbeat Bodyguard 2 device to the chest by electrodes designed for long-term measurements (Ambu A/S, type: L-00-S/25); one electrode will be attached to the right side of the body, immediately below to collarbone. The second electrode will be attached to the left side of the body, at the level of the rib cage. The Firstbeat device will be attached to the electrode below the collarbone and from the device a cable will connect to the electrode on the rib cage.

Raw R-R heartbeat intervals will be processed using Kubios HRV Premium 3.0.2. including algorithms for beat detection and artifact correction for each data file and subsequent calculation of HRV time-, −frequency and non-linear domain summary data [37, 38].

#### Salivary cortisol awakening response

A distinct component of diurnal cortisol rhythm in healthy humans is the morning rise in cortisol, which is a steep rise in cortisol during the 45 min following awakening. When the pattern of morning cortisol rise deviates from what is normally observed, it is argued that this is reflective of malfunction in neuroendocrine systems [39].

As recommended as a minimum for measurement of the cortisol awakening response, adult participants will collect saliva samples immediately upon awakening, 30 min and 45 min following awakening [39]. The saliva samples will be collected using Salivette®, code blue (Sarstedt), which contains a swab that absorbs saliva during chewing. Immediately at awakening, the participants will deliver the first saliva sample. Participants are instructed to not remove the electrodes and sleep apparatus (described earlier) until after the first saliva sample has been collected, such that awakening time can be assessed. Also, at awakening, the participants will start a dual timer (S. Brannan & Sons Ltd., England), which is set to count down from 30 and 45 min, simultaneously. When the timers reach 0, a distinct alarm tone rings notifying the participants that they must collect a second and third saliva sample, respectively. According to expert consensus it is recommended that caffeinated and sugared drinks, as well as food/breakfast is not consumed over the measurement period in the morning. Although brushing one’s teeth during cortisol saliva sampling is allowed [39], we have chosen a more conservative approach where we disallow this 10–15 min before each saliva sample. After each sample is delivered, the participants must place the sample in their household freezer.

In a checklist (described later) participants must report the exact time at which a saliva sample has been delivered. Also, the participants are handed a series of pairs of barcoded stickers, which contain a unique identifier for the person and the sample. After each sample, one sticker must be put in the personal checklist in a section indicating which saliva sample has be taken (day and time), whereas the other is put onto the Salivette containing the sample. In the checklist, also time of saliva sampling is reported by the subject.

At the final meeting, the samples will be transported in a freezer box to the Department of Sports Science and Clinical Biomechanics at the University of Southern Denmark. Here, they will be stored at − 20 degrees Celsius. When enough samples have been transported to the Department (no more than 500 samples), they will be transported in a freezer box from the University to the Clinical Biochemical Department at Slagelse Hospital (~ 70 km drive) for analysis for cortisol and cortisone content using the LC-MS method (Phenomenex Inc., USA, application 20,655). Before analysis, the samples are stored in the laboratory freezer at − 80 degrees Celcius. The laboratory has external quality control of their chemical analyses of salivary cortisol and cortisol by UK-NEQAS, England and are ranked satisfactory. The research staff will have continued dialogue with biochemists at the laboratory to assure that protocols for correct storage and analysis are met. Following laboratory analysis, the samples will be destroyed.

#### Salivary diurnal cortisol

Diurnal cortisol is composed of multiple measurable components of cortisol dynamics; first, as described above is the cortisol awakening response, and secondly, the decline of cortisol across the span of the day, i.e. the diurnal cortisol slope. In line with previous research [35], we will assess diurnal cortisol by adding a single saliva sample immediately before bedtime beyond the three saliva samples in the morning hours. By measuring cortisol at four points during daytime, we will estimate diurnal cortisol levels, to assess the state of the HPA axis output [35] during the span of the day.

#### Overview of questionnaires

In addition to the physical measurements we will also administer questionnaires at baseline and follow-up, to assess the effect of the screen media use restriction on different psychological and physical constructs. The adults will answer the following questionnaires; WHO-5, Profile of Moods Scale [40] and two components of the Leeds sleep evaluation questionnaire [41]. On behalf of the participating children, the adults will answer the Strength and Difficulties Questionnaire [42].

#### WHO-5

To assess the adult participants’ subjective sense of well-being we will administer the WHO-5 [43]. The tool consists of five simple and non-intrusive questions regarding an individual’s psychological well-being. The questionnaire has been translated into more than 30 languages and has been used in countless studies around the globe. WHO-5 has been demonstrated to be applicable in a wide array studies; it has been valuable tool in clinical studies, as well as a screening tool for depression. The clinometric validity of WHO-5 has been shown to be high [43].

#### Leeds sleep evaluation questionnaire

To assess subjective sense of restlessness during sleep and degree of interrupted sleep, we will use two of the 10 items of the Leeds Sleep Evaluation Questionnaire. The questionnaire items are answered on a visual analogue scale. The tool in its entirety has shown to be valid in some populations, both in healthy and in diseased individuals [41].

#### Profile of mood states

The adults’ mood state will be assessed using the original 64-item Profile of Moods States questionnaire, which is comprised of six scales relating to 6 states or emotions. The questionnaire has been used in psychological research for decades and has shown high internal consistency and construct validity for its mood scales [40].

#### Strengths and difficulties questionnaire

Data on Children’s wellbeing and mental health will be collected using the Danish parent reported Strength and Difficulties Questionnaire (SDQ) including the one-month follow-up versions, covering the age band 2–4, 5–6, 4–10 and 11–17 years adapted to kindergarten or school conditions. Parent reported SDQ has in general provided good psychometric properties [44, 45], also reported for Danish children [42]. Besides the total SDQ score and daily function, the broader internalising, externalising and prosocial subscales [46, 47] will be of primary interest in this low-risk population study.

### Objective monitoring of screen time: television, smartphone, tablet and computer activity

As shown in Fig. 3, throughout the entire course of the SCREENS trial we will objectively measure screen time on the participating families’ devices, including portable devices, such as smartphones and tablets, as well as televisions and computers.

#### SDU device tracker: monitoring of smartphone, tablet and personal computer activity

We have developed a non-commercial monitoring device (SDU Device Tracker) for monitoring screen time activity on smartphones, tablets and personal computers. For tablets and smartphones, the software is installed as an application using a custom-made QR-code. The app can track screen time activity, as well as the amount of times a device has been picked-up (opened). The app registers data on a second-to-second basis, thus allowing for detailed analysis of timing and amount of screen media use. We have developed a Python-based (Python 3.7.3) data reduction software, which perform data quality control and can summarize individual app data. Based on the processing of the data diagnostics of the application activity can be made, including how many times the app has been closed (force quitted) by the user and for how long, thus quantifying the amount of time where screen media use has not been recorded. Data from the application is continuously sent encrypted to a secure server at the University of Southern Denmark. The application is currently compatible with iOS and Android systems (smartphones and tablets) and OS X and Windows (PCs).

Although SDU Device Tracker will be installed on most devices, it may only be installed on devices where there is a possibility that said device will be used by a family member. Families may own several devices, including devices that they no longer use, e.g. devices stored in their basement. Therefore, due to time constraints during scheduled meetings, we may not install SDU Device Tracker on unused devices. However, we will take a conservative approach and install the application on most devices, where there is a small chance that said device might be used. For devices used for work, we may not be permitted to install the application.

The SDU Device Tracker applications have undergone extensive internal validation and quality control and we are currently conducting ongoing formal validation of the apps.

#### TV-monitoring device

A small electronic device was developed by engineers at our department (Department of Sports Science and Clinical Biomechanics) to assess the amount of TV usage. Detecting TV usage is assessed by measuring the power cord current using a hall sensor. A hall sensor is a current sensor generating a voltage proportional to the current flowing through the sensor. The voltage is converted using an analog to digital converter with the installed micro-controller, in 1-min epocs. TV usage is detected by using a simple threshold, which is set substantial higher than the stand-by current (equal to or above the signal strength half-way between the minimum and maximal). By using this threshold, we also consider the fact that some TVs exhibit short burst of electrical activity, often during nocturnal hours. These signals, which are multiples above standby signal strength, but also multiples below television activity (which is often a 5-fold signal stronger than stand-by mode). The signals are characteristic of TV, which download information for the TV-users, when the TV is shut off. By using the defined cut-off above, TV-time is easily distinguished from these shorts burst of electrical activity not associated with TV usage.

At baseline, we hand out a television usage checklist, which we place by each television with more than one user. Each checklist will contain a page for each day of the baseline period. Here, each family member must mark whether they have used the television in 15-min intervals of the day (03:00–03:00). It is possible for family members to mark the same time slots. The marked time slots will then be cross-referenced with the objective measures of tv-usage to categorize individual TV usage on shared TVs.

We may refrain from installing a TV-monitor on televisions that may never be used, e.g. TVs tucked away in the basement, which may not even be plugged in.

### Sheets and personalized checklists

#### Diaries

During the intervention, the subjects must fill out small sheets. First, every member of the family must report the amount and type of entertainment-based screen time that they have used, of the up to three hours per week, which is permitted. Each family member is handed their own personal sheet to fill in these details. Secondly, the subjects are asked to report, on a separate paper, if they have exceeded the limit for entertainment-based screen media use. In this sheet, any screen media use of ‘passive’ participants, is also reported. Third, for those participants for whom screen time is necessary during everyday life, i.e. during school- or work life, a third sheet is handed out. On this sheet this necessary screen time during the 2-week experiment period must be noted.

#### Personalized checklists

Each participant will receive a detailed but concise personal checklist, which chronicles the content of the baseline and follow-up days in terms of the respective measurement protocols. Each checklist contains the dates and times which are relevant to the physical measurements, including e.g. when devices should be worn, when existing electrodes must be switched to new ones and when devices must be removed. As described earlier, adult participants must also register the number and timing of the saliva samples that they have collected. Participants must also register any irregularities pertaining to the physical measurements, e.g. issues with timing and reasons for non-wear. Lastly, in the checklist participants must also when they wake up, when they go to work or school (and if they are sick), when they leave work or school, as well as when they go to bed. This information will also be used to time annotate the time series data.

### Compliance

To estimate the degree to which families are compliant to the intervention two calculations will be made for each family: 1) a calculation of a threshold for compliance for entertainment-based screen time and 2) an estimate of total entertainment-based screen time during the intervention. The threshold for compliance will equal the maximum entertainment-based screen time permitted. The threshold is calculated by multiplying the number of participating family members by 3 h/week of permitted screen time and multiplying this by 2 weeks, i.e. the duration of the experiment.

$$ \mathbf{Threshold}\ \mathbf{for}\ \mathbf{family}-\mathbf{wise}\ \mathbf{compliance}=\mathrm{n}\ \mathrm{participating}\ \mathrm{family}\ \mathrm{members}\times 3\kern0.28em \mathrm{hours}/\mathrm{week}\times 2\kern0.28em \mathrm{weeks} $$

In this calculation it is assumed that each family member uses their permitted screen time by themselves. As family members may use their entertainment-based screen time together, we expect that a compliant family will have a level of entertainment-based screen time lower than the compliance threshold.

To estimate the total amount of entertainment-based screen time, multiple sources of data will be used. First, objectively measured TV-, smartphone-, tablet-, ipad- and PC-time will be summarized. Then, entertainment-based screen time by self-report, on devices whose activity we do not measure, e.g. TV-viewing on a TV outside the household, will be added to the summary of objectively assessed data. Next, we will subtract all self-reported necessary screen time on devices that we measure, which otherwise would be considered entertainment based. Lastly, we will subtract all screen time (entertainment-based and necessary) by ‘passive’ participants.

$$ \mathbf{Family}-\mathbf{wise}\ \mathbf{level}\ \mathbf{of}\ \mathbf{entertainment}-\mathbf{based}\ \mathbf{screen}\ \mathbf{time}=\mathrm{total}\ \mathrm{objective}\mathrm{ly}\ \mathrm{measured}\ \mathrm{screen}\ \mathrm{time}\ \left(\min /2\ \mathrm{weeks}\right)+\mathrm{self}-\mathrm{reported}\kern0.6em \mathrm{entertainment}-\mathrm{based}\ \mathrm{screen}\ \mathrm{time}\ \mathrm{beyond}\ \mathrm{objective}\ \mathrm{measures}\ \left(\min /2\ \mathrm{weeks}\right)-\mathrm{self}-\mathrm{reported}\ \mathrm{necessary}\ \mathrm{screen}\ \mathrm{time}\ \left(\min /2\ \mathrm{weeks}\right)\ \mathrm{in}\ \mathrm{objective}\ \mathrm{measures}-\mathrm{self}-\mathrm{reported}\ \mathrm{screen}\ \mathrm{time}\ \mathrm{in}\ {\mathrm{objective}\ \mathrm{measures}}_{\mathrm{passive}\ \mathrm{participants}}\ \left(\min /2\ \mathrm{weeks}\right) $$

To compute degree of compliance for a family, we will calculate the proportion of entertainment-based screen time of the compliance threshold, where a number below or equal to 1 or 100% will indicate that the family have been compliant to the intervention protocol.

We will also attempt to calculate individual level compliance using all the available data sources. However, this computation may be complicated by the fact that it may not always be clear who the user of a shared device is. For this reason, it may be difficult to parse all screen media use in a household onto distinct users. Also, although we will attempt to describe individual usage on shared televisions based on television usage checklists filled out during baseline (described earlier), we cannot be certain that tv usage profile during baseline can be generalized to the remainder of the study period.

### Questionnaire: feasibility of and compliance to the intervention

At the end of the intervention, each adult must complete a questionnaire concerning; 1) the degree to which the adult was compliant to the intervention protocol, 2) the challenges and opportunities the family faced during the 2-week screen time reduction period and 3) the changes that were made during everyday life during the period of the intervention. Questions relating to the latter will mainly be concerned with what activities that were introduced or emphasized, when screen time was no longer possible to the same extent. One of the adults must also complete a similar questionnaire on behalf of the children.

### Background data

Basic background information is collected in the survey, including educational attainment according to the International Standard Classification of Education, work experience and current employment, as well as household constellation. Data on ethnicity (ethnic origin) is gathered from the Danish Civil Registry and is available for the selected children and selected adults, as well as other parents of the child not registered at the same address. From the survey and initial meetings with the families, data on screen time habits and culture within the family household regarding screen time is collected. Beyond the amount and timing of screen time, data on the number of devices in the household, the age when the child had his/her own smartphone, rules regarding screen media use and screen media culture around family meals, among others, is also collected. At baseline, each adult on behalf of themselves and their child must report their current bodyweight (kg) and height (cm) to compute body mass index. Also, data on gender and age will be collected for all participants.

### Data safety

Survey data will be collected and stored using REDCap (a secure application for building and managing online surveys and databases), which is managed by the researcher service organization OPEN in the Region of Southern Denmark. This mode of storage is in accordance with the General Data Protection Regulation for data handling. Exposure and outcome data collected during the trial will be stored in the families’ households until completion of the study, after which it will be transported back to the University of Southern Denmark. At the University, data will be extracted from the measurement devices and stored in safe folders on the University servers.

Currently MGR, JP, LGO, PLK, JCB, AG and our pre-graduate research scholar and two student helpers have been granted access to the data by the Danish Data Protection Agency. The data will be stored in its raw form and no statistical investigation will be conducted, before the data collection is complete.

## Statistical methods

A detailed description of data management and our a priori planned statistical analyses can be found at (see 'Study documents'): https://clinicaltrials.gov/ct2/show/NCT04098913?cond=screens&draw=2&rank=1.

## Discussion

This paper describes in detail the protocol for a randomized controlled trial that aims to investigate the short-term efficacy of limiting leisure screen media use on objectively assessed habitual physical activity, sleep, and physiological stress in parents and their 4–14-year old children, in free-living.

The SCREENS trial addresses a research gap and limitations of observational and experimental studies of the effects of screen media use in children and adults. This includes possible lack of generalizability of experimental laboratory findings to free-living conditions, that may limit findings from acute effect cross-over studies of exposure to digital screen devices during evening hours and sleep [18, 19, 48, 49], and of acute effects of availability of screen devices on physical activity behavior [50]. The study also attempts to overcome limitations from experimental studies conducted in free-living that have not documented compliance to screen media during intervention precisely, or have not minimized noncompliance [12, 14]. In our study we will attempt to objectively monitor compliance by installing newly developed monitoring systems on screen time devices in the participating families’ households. This will markedly decrease the reliance on memory and influence of social desirability bias in gathering compliance data on screen media use. Also, we have taken important steps in the enrollment phase (e.g. exclusion based on parents’ perceived resources for behavior change) to enhance the families’ compliance to the screen media restriction protocol and during the intervention by closely following the families and optimizing the participants’ experience. In an efficacy trial conducted in free-living condition as the present, these aspects are essential to make valid causal inferences from the data. Also, importantly, much of the available research on screen time and health is, for obvious historical reasons, not based on screen media use in its modern form, but rather television usage, and is also to a large extent based on self-report [7].

Our goal is that results from statistical analyses will move the field forward towards answering causal questions which currently remain unanswered. Furthermore, registration of screen time activity is collected in no higher than 1-min intervals, and opportunities for investigating with high accuracy and detail, not only how amount but also timing of screen time exposure is related to parameters of health, will be rendered possible in secondary exploratory analyses. By merging objective data on screen time consumption, with high-quality 24-h measurements on highly detailed accelerometry and heart-rate-variability, as well as detailed EEG data during nocturnal hours, we will generate a data resource from which detailed investigations are possible. Also, by conducting the study in a familiar setting, i.e. in the families’ home during everyday life and not in a laboratory, the ecological validity of the results will be high.

We hope that the findings from this study, will help advance the research frontier within the field of screen time and health. Importantly, it is our vision that the results from the SCREENS trial, alongside results from other new studies in the field, will provide a solid foundation for policy makers to make political decisions regarding usage of screen-based media, e.g. formulate concrete national guidelines or recommendations for screen media use in children, adolescents and adults. Also, as we expect that our findings will be of public interest, the results will be disseminated to practitioners in relevant fields, including practitioners in municipal settings as well as at institutions in the private sector, whose work revolves around childhood health. Also, results will be presented at scientific conferences nationally and internationally and published in peer-reviewed journals (regardless of the direction of the findings). Our work may inspire our scientific colleagues in their future work, including replication of our study in other national and cultural settings, as well as developing intervention studies, whose aims are to facilitate long-term behavior change regarding screen time for specific populations who consume screen media in excessive amounts.

## Supplementary information

**Additional file 1.** SPIRIT_checklist.doc’. The additional file includes the SPIRIT checklist for study protocols of clinical trials, regarding the SCREENS trial. An indication of where we have addressed each item is noted in the checklist.

## Data Availability

Upon completion of the data collection process and when structured datasets have been developed, the data may be made available for use beyond the research staff involved. Availability of the data and the material, including coding and material, e.g. consent forms and checklist templates, used in the data collection process, can be made available upon application to the head of research and project leader of the SCREENS trial Professor Anders Grøntved (agroentved@health.sdu.dk), as well following upon approval from the Danish Data Protection Agency, if necessary.

## Abbreviations

HRV: Heart rate variability
OPEN: Odense patient data explorative network
