# Supplementary material for: Short-term efficacy of reducing screen media use on physical activity, sleep, and physiological stress in families with children aged 4–14: study protocol for the SCREENS randomized controlled trial
Source: BMC Public Health. 2020 Mar 23;20:380. doi: 10.1186/s12889-020-8458-6 (PMC7092494; doi:10.1186/s12889-020-8458-6)
Supplement: Supplementary file 1 — Additional file 1. SPIRIT_checklist.doc’. The additional file includes the SPIRIT checklist for study protocols of clinical trials, regarding the SCREENS trial. An indication of where we have addressed each item is noted in the checklist. [file 12889_2020_8458_MOESM1_ESM.doc]

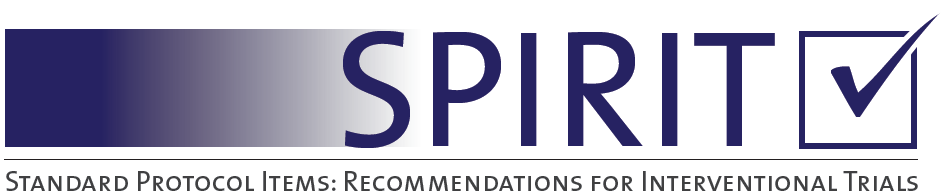


| Section/item | Item no | Description | Note on where this item no. is addressed |
| --- | --- | --- | --- |
| **Administrative information** | | |  |
| Title | 1 | Descriptive title identifying the study design, population, interventions, and, if applicable, trial acronym | Addressed in title on page 1 |
| Trial registration | 2a | Trial identifier and registry name. If not yet registered, name of intended registry | Noted under abstract |
|  | 2b | All items from the World Health Organization Trial Registration Data Set | In the interest of limiting the amount of text, this is not listed in the manuscript, but rather it is included in the registration at Clinicaltrials.gov, which can be found by clicking the link below: <https://clinicaltrials.gov/ct2/show/NCT04098913> |
| Protocol version | 3 | Date and version identifier | Is addressed in a separate document: “Study protocol amendment versions.docx” |
| Funding | 4 | Sources and types of financial, material, and other support | This item is addressed in the section ‘Funding’ immediately preceding the ‘Authors' contributions’ section |
| Roles and responsibilities | 5a | Names, affiliations, and roles of protocol contributors | Names and affiliations are listed below the title. Protocol contributions is addressed in the ‘Authors' contributions’ section |
|  | 5b | Name and contact information for the trial sponsor | The principal investigator may be contacted via the e-mail specified in the ‘Availability of data and material’ section |
|  | 5c | Role of study sponsor and funders, if any, in study design; collection, management, analysis, and interpretation of data; writing of the report; and the decision to submit the report for publication, including whether they will have ultimate authority over any of these activities | Addressed in the ‘Funding’ section |
|  | 5d | Composition, roles, and responsibilities of the coordinating centre, steering committee, endpoint adjudication committee, data management team, and other individuals or groups overseeing the trial, if applicable (see Item 21a for data monitoring committee) | Addressed in the ‘Organization of the SCREENS trial’ section |
| Introduction |  |  |  |
| Background and rationale | 6a | Description of research question and justification for undertaking the trial, including summary of relevant studies (published and unpublished) examining benefits and harms for each intervention | Addressed in ‘Background’ |
|  | 6b | Explanation for choice of comparators | Addressed in the ‘Intervention’ section |
| Objectives | 7 | Specific objectives or hypotheses | Addressed in the ‘Background’ and ‘Objectives’ sections |
| Trial design | 8 | Description of trial design including type of trial (eg, parallel group, crossover, factorial, single group), allocation ratio, and framework (eg, superiority, equivalence, noninferiority, exploratory) | Addressed several places. Already in the ‘Background’ section it is specified that the intervention is evaluated using a randomized controlled trial design. |
| Methods: Participants, interventions, and outcomes | | |  |
| Study setting | 9 | Description of study settings (eg, community clinic, academic hospital) and list of countries where data will be collected. Reference to where list of study sites can be obtained | Addressed in abstract (Country) and ‘SCREENS survey and randomized controlled trial’ and ‘Recruitment process’ sections |
| Eligibility criteria | 10 | Inclusion and exclusion criteria for participants. If applicable, eligibility criteria for study centres and individuals who will perform the interventions (eg, surgeons, psychotherapists) | Addressed in “Stage 1: Recruitment via survey” (survey-level criteria) and “Stage 2: Recruitment following survey” (trial level criteria) |
| Interventions | 11a | Interventions for each group with sufficient detail to allow replication, including how and when they will be administered | Addressed in “Intervention” section |
|  | 11b | Criteria for discontinuing or modifying allocated interventions for a given trial participant (eg, drug dose change in response to harms, participant request, or improving/worsening disease) | Addressed in the “Participant safety” section |
|  | 11c | Strategies to improve adherence to intervention protocols, and any procedures for monitoring adherence (eg, drug tablet return, laboratory tests) | Improve adherence:  Addressed in the ‘Theoretical underpinning of the intervention’ section  Monitor adherence:  Addressed in ‘SDU Device Tracker: Smartphones, tablets and personal computers’, ‘Tv-monitoring device’ and ‘Questionnaire: feasibility of and compliance to the intervention’ |
|  | 11d | Relevant concomitant care and interventions that are permitted or prohibited during the trial | Not deemed relevant |
| Outcomes | 12 | Primary, secondary, and other outcomes, including the specific measurement variable (eg, systolic blood pressure), analysis metric (eg, change from baseline, final value, time to event), method of aggregation (eg, median, proportion), and time point for each outcome. Explanation of the clinical relevance of chosen efficacy and harm outcomes is strongly recommended | Addressed in the ‘Primary and secondary outcomes and endpoints’ section  Clinical relevancy addressed in ‘Justification of sample size’ section |
| Participant timeline | 13 | Time schedule of enrolment, interventions (including any run-ins and washouts), assessments, and visits for participants. A schematic diagram is highly recommended (see Figure) | Addressed in the ‘SCREENS survey and randomized controlled trial’ and ‘Recruitment process’ sections, as well as in Figure 1. |
| Sample size | 14 | Estimated number of participants needed to achieve study objectives and how it was determined, including clinical and statistical assumptions supporting any sample size calculations | Addressed in the ‘Justification of sample size’ section |
| Recruitment | 15 | Strategies for achieving adequate participant enrolment to reach target sample size | Addressed in the ‘Stage 1: Recruitment via survey’ section |
| **Methods: Assignment of interventions (for controlled trials)** | | |  |
| Allocation: |  |  |  |
| Sequence generation | 16a | Method of generating the allocation sequence (eg, computer-generated random numbers), and list of any factors for stratification. To reduce predictability of a random sequence, details of any planned restriction (eg, blocking) should be provided in a separate document that is unavailable to those who enrol participants or assign interventions | Addressed in ‘Post-baseline/pre-experiment meeting and randomization’ section |
| Allocation concealment mechanism | 16b | Mechanism of implementing the allocation sequence (eg, central telephone; sequentially numbered, opaque, sealed envelopes), describing any steps to conceal the sequence until interventions are assigned | Also addressed in ‘Post-baseline/pre-experiment meeting and randomization’ section |
| Implementation | 16c | Who will generate the allocation sequence, who will enrol participants, and who will assign participants to interventions | Also addressed in ‘Post-baseline/pre-experiment meeting and randomization’ section |
| Blinding (masking) | 17a | Who will be blinded after assignment to interventions (eg, trial participants, care providers, outcome assessors, data analysts), and how | Also addressed in ‘Post-baseline/pre-experiment meeting and randomization’ section |
|  | 17b | If blinded, circumstances under which unblinding is permissible, and procedure for revealing a participant’s allocated intervention during the trial | Not relevant given 17a |
| **Methods: Data collection, management, and analysis** | | |  |
| Data collection methods | 18a | Plans for assessment and collection of outcome, baseline, and other trial data, including any related processes to promote data quality (eg, duplicate measurements, training of assessors) and a description of study instruments (eg, questionnaires, laboratory tests) along with their reliability and validity, if known. Reference to where data collection forms can be found, if not in the protocol | Addressed separately for each outcome in the ‘Outcome measures’ section |
|  | 18b | Plans to promote participant retention and complete follow-up, including list of any outcome data to be collected for participants who discontinue or deviate from intervention protocols | No additional data will be collected on participants who discontinue or deviate from protocol. However, sensitivity analyses will be conducted on survey data (addressing selection bias). |
| Data management | 19 | Plans for data entry, coding, security, and storage, including any related processes to promote data quality (eg, double data entry; range checks for data values). Reference to where details of data management procedures can be found, if not in the protocol | Addressed in ‘Data Management and statistical analyses plan (SAP)’ published at: <https://clinicaltrials.gov/ct2/show/NCT04098913>  Addressed briefly, however, in ‘Data safety’ and throughout ‘Outcome measures’ section |
| Statistical methods | 20a | Statistical methods for analysing primary and secondary outcomes. Reference to where other details of the statistical analysis plan can be found, if not in the protocol | Also addressed in ‘Data Management and statistical analyses plan (SAP)’ |
|  | 20b | Methods for any additional analyses (eg, subgroup and adjusted analyses) | Also addressed in ‘Data Management and statistical analyses plan (SAP)’ |
|  | 20c | Definition of analysis population relating to protocol non-adherence (eg, as randomised analysis), and any statistical methods to handle missing data (eg, multiple imputation) | Also addressed in ‘Data Management and statistical analyses plan (SAP)’ |
| **Methods: Monitoring** | | |  |
| Data monitoring | 21a | Composition of data monitoring committee (DMC); summary of its role and reporting structure; statement of whether it is independent from the sponsor and competing interests; and reference to where further details about its charter can be found, if not in the protocol. Alternatively, an explanation of why a DMC is not needed | No data monitoring committee will be established, as the amount of data that will be collected will be done in accordance with that estimated by the power calculation. Data will be left in its raw form until trial completion, as al |
|  | 21b | Description of any interim analyses and stopping guidelines, including who will have access to these interim results and make the final decision to terminate the trial | No interim analyses will be conducted. No termination criteria have been set up, although participants can withdraw from the study at any time. |
| Harms | 22 | Plans for collecting, assessing, reporting, and managing solicited and spontaneously reported adverse events and other unintended effects of trial interventions or trial conduct | Not deemed relevant, although it will be noted if there are adverse events or harms that the researchers are unaware of |
| Auditing | 23 | Frequency and procedures for auditing trial conduct, if any, and whether the process will be independent from investigators and the sponsor | No trial auditing will be conducted |
| Ethics and dissemination | | |  |
| Research ethics approval | 24 | Plans for seeking research ethics committee/institutional review board (REC/IRB) approval | Addressed in ‘Ethics approval and consent to participate’ |
| Protocol amendments | 25 | Plans for communicating important protocol modifications (eg, changes to eligibility criteria, outcomes, analyses) to relevant parties (eg, investigators, REC/IRBs, trial participants, trial registries, journals, regulators) | Also addressed in the ‘Ethics approval and consent to participate’ section |
| Consent or assent | 26a | Who will obtain informed consent or assent from potential trial participants or authorised surrogates, and how (see Item 32) | Also addressed in the ‘Ethics approval and consent to participate’ section |
|  | 26b | Additional consent provisions for collection and use of participant data and biological specimens in ancillary studies, if applicable | Not relevant |
| Confidentiality | 27 | How personal information about potential and enrolled participants will be collected, shared, and maintained in order to protect confidentiality before, during, and after the trial | Addressed in statistical analysis plan (SAP) published at: <https://clinicaltrials.gov/ct2/show/NCT04098913> |
| Declaration of interests | 28 | Financial and other competing interests for principal investigators for the overall trial and each study site | Addressed in ‘Competing interests’ |
| Access to data | 29 | Statement of who will have access to the final trial dataset, and disclosure of contractual agreements that limit such access for investigators | Addressed in ‘Data safety’ |
| Ancillary and post-trial care | 30 | Provisions, if any, for ancillary and post-trial care, and for compensation to those who suffer harm from trial participation | Not relevant |
| Dissemination policy | 31a | Plans for investigators and sponsor to communicate trial results to participants, healthcare professionals, the public, and other relevant groups (eg, via publication, reporting in results databases, or other data sharing arrangements), including any publication restrictions | Addressed in ‘Discussion’ |
|  | 31b | Authorship eligibility guidelines and any intended use of professional writers | Addressed in the ‘Authors' contributions’ section |
|  | 31c | Plans, if any, for granting public access to the full protocol, participant-level dataset, and statistical code | Addressed in the ‘Availability of data and material’ section |
| Appendices |  |  |  |
| Informed consent materials | 32 | Model consent form and other related documentation given to participants and authorised surrogates | Also addressed in the ‘Availability of data and material’ section |
| Biological specimens | 33 | Plans for collection, laboratory evaluation, and storage of biological specimens for genetic or molecular analysis in the current trial and for future use in ancillary studies, if applicable | Addressed in the ‘Salivary Cortisol Awakening Response’ section |

*It is strongly recommended that this checklist be read in conjunction with the SPIRIT 2013 Explanation & Elaboration for important clarification on the items. Amendments to the protocol should be tracked and dated. The SPIRIT checklist is copyrighted by the SPIRIT Group under the Creative Commons “[Attribution-NonCommercial-NoDerivs 3.0 Unported](http://www.creativecommons.org/licenses/by-nc-nd/3.0/)” license.
